# Influence of pH, bleaching agents, and acid etching on surface wear of bovine enamel

**DOI:** 10.1590/1678-775720150281

**Published:** 2016

**Authors:** Ana Flávia Soares, Juliana Fraga Soares Bombonatti, Marina Studart Alencar, Elaine Cristina Consolmagno, Heitor Marques Honório, Rafael Francisco Lia Mondelli

**Affiliations:** 1 - Universidade de São Paulo, Faculdade de Odontologia de Bauru, Departamento de Dentística, Endodontia e Materiais Odontológicos, Bauru, SP, Brasil.; 2 - Universidade de São Paulo, Faculdade de Odontologia de Bauru, Departamento de Odontopediatria, Ortodontia e Saúde Coletiva, Bauru, SP, Brasil.

**Keywords:** Tooth bleaching, Dental acid etching, Hydrogen-ion concentration, Tooth wear

## Abstract

Development of new materials for tooth bleaching justifies the need for studies to evaluate the changes in the enamel surface caused by different bleaching protocols.

**Objective:**

The aim of this study was to evaluate the bovine dental enamel wear in function of different bleaching gel protocols, acid etching and pH variation.

**Material and Methods:**

Sixty fragments of bovine teeth were cut, obtaining a control and test areas. In the test area, one half received etching followed by a bleaching gel application, and the other half, only the bleaching gel. The fragments were randomly divided into six groups (n=10), each one received one bleaching session with five hydrogen peroxide gel applications of 8 min, activated with hybrid light, diode laser/blue LED (HL) or diode laser/violet LED (VHL) (experimental): Control (C); 35% Total Blanc Office (TBO35HL); 35% Lase Peroxide Sensy (LPS35HL); 25% Lase Peroxide Sensy II (LPS25HL); 15% Lase Peroxide Lite (LPL15HL); and 10% hydrogen peroxide (experimental) (EXP10VHL). pH values were determined by a pHmeter at the initial and final time periods. Specimens were stored, subjected to simulated brushing cycles, and the superficial wear was determined (μm). ANOVA and Tukey´s tests were applied (α=0.05).

**Results:**

The pH showed a slight decrease, except for Group LPL15HL. Group LPS25HL showed the highest degree of wear, with and without etching.

**Conclusion:**

There was a decrease from the initial to the final pH. Different bleaching gels were able to increase the surface wear values after simulated brushing. Acid etching before bleaching increased surface wear values in all groups.

## INTRODUCTION

Among the techniques to reestablish the good aesthetics for discolored vital teeth, vital tooth bleaching is a more conservative and less invasive alternative than direct and indirect conventional restoration techniques and has been well accepted among patients because they consider it a safe and effective technique^22^. The effectiveness of dental bleaching is influenced by several factors, such as the bleaching system used, bleaching agent concentration, application time and the application of light^1^ ^,^ ^16^ ^,^ ^26^.

Light sources can be used to optimize the bleaching procedure in an attempt to increase the rates of peroxide dissociation and reduce the bleaching session^2^ ^,^ ^7^ ^,^ ^16^. Analyzing the bovine enamel wear caused by different bleaching techniques, the use of a light source did not influence the tooth enamel wear because similar results were obtained with and without light sources^13^ ^,^ ^23^. In addition to the above-mentioned factors, the pH of the bleaching agents^19^ and acid etching before treatment^6^ ^,^ ^12^ ^,^ ^16^ ^,^ ^25^ are also factors that can directly influence the whitening process. At the present, manufacturers have shown concern about the acidity of bleaching gels because a pH below the critical point (pH 5.5 to 6.5)^21^ may result in the dissolution of enamel, microhardness alterations^14^, increased sensitivity, increased enamel wear and surface roughness^13^ ^,^ ^23^.

In an endeavor to control demineralization, which is able to increase the penetration of the bleaching gel and, consequently, tooth bleaching^16^, the acid etching technique can be used before the bleaching procedure^6^. This technique works by producing changes in the enamel surface in two distinct ways. First, the acid removes the surface layer and, consequently, the plaque and acquired film are removed and, second, the remaining enamel with a more porous surface layer is also removed^20^. A greater ability for peroxide penetration was obtained when the previous acid etching was performed with a light source^6^. An increasing number of tooth-bleaching products are commercially available with different compositions and concentrations^11^, and nowadays, there is a trend towards decreasing the gels’ concentration values and diversifying their formulations^1^ ^,^ ^3^ ^,^ ^23^ ^,^ ^24^. The development of these new materials justifies the need for studies to evaluate the changes in the enamel surface caused by different bleaching protocols.

Thus, the aim of this *in vitro* study was to evaluate the bovine dental enamel wear in function of different bleaching gel protocols, the acid etching and pH variation.

## MATERIAL AND METHODS

**Figure 1:**
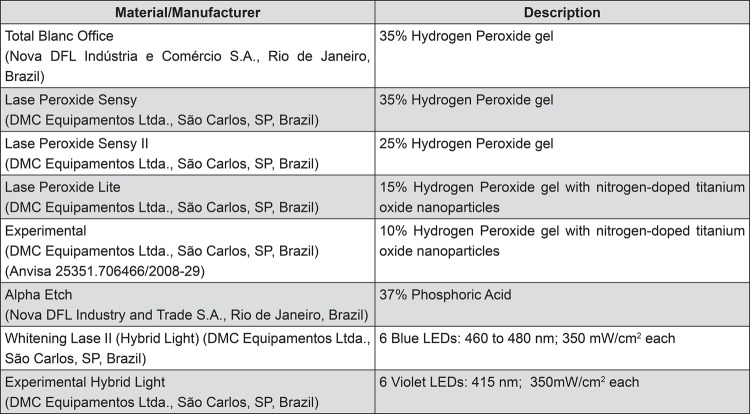
Materials and equipment used in this study

This is an *in vitro* study, and the experimental design for pH analisys was divided into two study factors: bleaching agent [in five levels: bleaching agents based on hydrogen peroxide (H_2_O_2_), two at 35%, one at 25%, one at 15%, and one at 10%] and time periods (at two levels: before and after bleaching). Wear analysis consisted of one study factor: treatment [in 11 levels: Control (did not receive the acid etching and bleaching gel), and two at H_2_O_2_ 35%, one at 25%, one at 15%, and one at 10%, with or without acid etching]. Different light sources employed were not classified as study factors because different bleaching treatments use different light sources following the protocol indicated by the manufacturer. The control group and five experimental groups are described in Figure 1.

### Material

The materials and equipments used for this study are outlined in Figure 1.

### Methods

#### Specimen preparation

**Figure 3:**
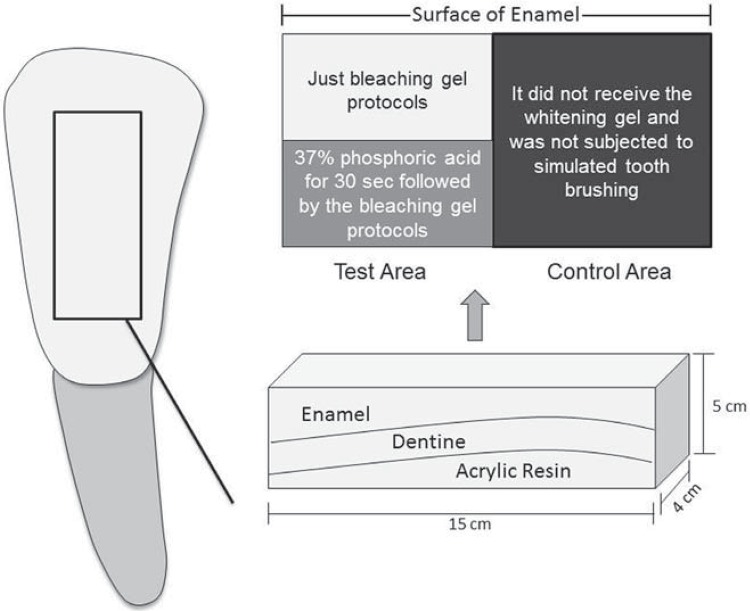
Division of the specimen, demonstrating the test (with and without acid conditioning) and control area

A total of 60 bovine mandibular central incisors were chosen for the study. They were cut into rectangular shapes and embedded in slow-setting acrylic resin blocks (15x5x4 mm). A flat enamel surface was produced by wet polishing with 320, 400, 600 and 1200-grit silicon carbide abrasive papers. To control the individual variation in the enamel surface characteristics among the tested teeth, each specimen was divided into two halves, providing a control area and a test area^13^ ^,^ ^18^ ^,^ ^23^. The test area was further divided, with one-half receiving 37% phosphoric acid (Alpha Etch; Nova DFL Indústria e Comércio S.A., Rio de Janeiro, Brazil) for 30 seconds followed by the bleaching gel protocols, and the other half receiving only the bleaching gel protocols (Figure 3).

#### Bleaching procedure

**Figure 2:**
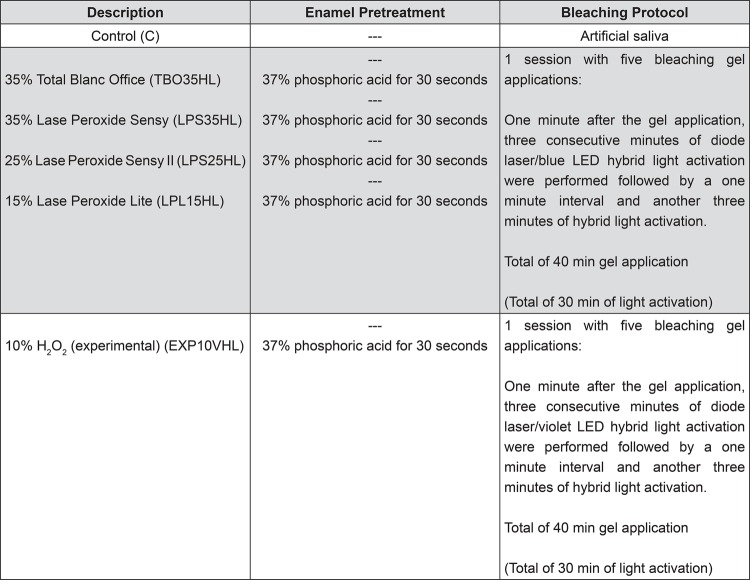
Groups and bleaching protocols used

The specimens were randomly divided into six groups (n=10) described in Figure 2.

#### Measurement of pH levels of bleaching gels

To analyze the pH levels of the bleaching gels, a portable pHmeter with a digital display (Model 1001 Sentron, Sentron, WA, USA) was used. This device has an electrode of small dimensions, compatible with the size of the specimen. It could make a readout with an accuracy of 0.01 in a few seconds and with minimal waste of bleaching gel. Before beginning each group of readouts, the pHmeter was calibrated with double standard substances (pH 4.0 and 7.0). The initial and final pH levels of the bleaching gels were obtained from the average of readings for each of the five applications of the bleaching gel to the tooth surface. The first readout was taken 30 seconds after the contact between the bleaching gel and the bovine enamel, and, the second readout, 30 seconds after the end of the time used, with activation with a hybrid light source^23^.

#### Brushing abrasion

All test areas, of the 60 studied specimens (divided into six groups), were subjected to simulated tooth brushing. The tooth-brushing machine remained in a controlled temperature of 37±2°C with soft nylon bristles (Colgate Classic CLEAN, Colgate Palmolive Industrial Ltda., Osasco, SP, Brazil), applying a load of 300 grams on the samples. Slurry was prepared by mixing 2:1 of deionized water and Colgate MFP (Colgate Palmolive, Osasco, SP, Brazil) dentifrice by weight immediately before testing. One hundred thousand brushing strokes were performed for each specimen and the bristles were changed at the half-cycle point. After testing, the specimens were rinsed under running water and cleaned in a sonic device (Cristófoli Biosafety Equipment Ltda., Londrina, PR, Brazil) in deionized water for 10 minutes, and then stored in fresh deionized water (37±2°C)^13^ ^,^ ^15^ ^,^ ^18^ ^,^ ^23^.

#### Wear determination

Wear was quantified by means of a roughness meter (Hommelwerke GmbH ref. #240851, Schwenningen, Germany) connected to a PC with a specific software program to provide the profiles of the surfaces tested. For each segment of the specimen, six measurements were carried out at random, three readouts were performed on each portion (with or without acid etching) and the mean wear value was calculated (Figure 3). These profilometric traces were taken from the reference surface (protected area) on one side to the opposite one, crossing the surface exposed to the experimental procedures. Thus, the wear readout was performed by the surface profile, measuring the distance in micrometers between the graph midline, corresponding to the specimen plane line (intact area) and the deepest valley corresponding to the ground area^8^ ^,^ ^13^ ^,^ ^18^ ^,^ ^23^.

## Statistical analysis

For correlation analyses between pH and wear, the Pearson correlation test was used. For pH analysis, two-way repeated measures ANOVA followed by the Tukey`s test were used. For wear, one-way ANOVA followed by the Tukey’s test were performed. The level of significance of 5% was adopted.

## RESULTS

There was no correlation between pH and wear. When analyzing the pH values (Table 1), a statistically significant decrease between the initial and final pH was noted, except in the 15% Lase Peroxide Lite activated with hybrid light group. The lowest values were found in the 35% hydrogen peroxide Groups activated with hybrid light at the end of the bleaching agent application. The group that showed more alkaline was the experimental 10% hydrogen peroxide Group with nitrogen-doped titanium oxide nanoparticles, activated with violet hybrid light, which had a lower concentration of hydrogen peroxide for both the initial and final measurements.

**Table 1:** Means and standard deviation of the initial and final pH values of the study groups

Groups	Time of measurement	Mean ± Standard Deviation*
TBO35HL	Initial Final	6.42±0.27^A^ 6.01±0.11^B^
LPS35HL	Initial Final	7.08±0.25^C^ 6.11±0.11^B^
LPS25HL	Initial Final	7.12±0.05^C^ 6.42±0.08^A^
LPL15HL	Initial Final	7.59±0.08^D^ 7.47±0.07^D^
EXP10VHL	Initial Final	9.23±0.05^E^ 8.99±0.07^F^

*Different capital letters indicate a statistically significant difference between lines, at the level of 5% calculated by the Tukey´s method

**Figure 4:**
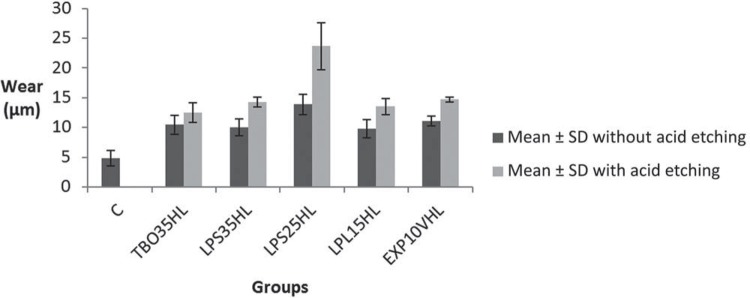
Graphic of mean (µm) and standard deviation of wear of the groups (C - without etching and without bleaching - and other groups with only bleaching or acid etching prior to bleaching)

The one-way ANOVA test for evaluating the surface wear showed statistically significant differences between the tested groups (p<0.05). Next, the Tukey’s test was applied for the values obtained (Table 2). All groups, except the 35% Total Blanc Office Group, had a statistically significant increase when acid etching was previously performed. The lowest wear value was found in control group (Group C), which did not undergo the acid etching and dental bleaching, and the 25% Lase Peroxide Sensy II Group (LPS25HL) with prior acid etching showed the highest wear value. The Group LPS25HL without acid etching showed similar results (p<0.05) to the other groups where the etching was done (Figure 4).

**Table 2:** Means (µm) and standard deviation of wear of the groups studied, comparing the two treatments [bleaching (B) and acid etching + bleaching (A)] and the control group

Groups	Mean ± Standard Deviation*
C (no bleaching)	4.86±1.28^A^
TBO35HLB	10.47±1.58^BD^
TBO35HLA	12.49±1.60^CDE^
LPS35HLB	9.97±1.42^BD^
LPS35HLA	14.21±0.83^C^
LPS25HLB	13.85±1.71^C^
LPS25HLA	23.67±3.93^F^
LPL15HLB	9.80±1.52^B^
LPL15HLA	13.51±1.38^CE^
EXP10VHLB	11.13 ±0.82^BDE^
EXP10VHLA	14.70±0.40^C^

*Different capital letters indicate a statistically significant difference between lines, at the level of 5% calculated by the Tukey´s method

## DISCUSSION

Most studies on dental bleaching investigates the morphological changes in the enamel^10^ ^,^ ^13^ ^,^ ^14^ ^,^ ^23^ and the pulp damage^5^ ^,^ ^17^ caused by different techniques, however, the changes caused by differences of the bleaching gels’ pH is still controversial^23^. In this study when observing the results obtained by pH analysis of bleaching agents, one could conclude that there was a trend towards a decrease in the mean pH values between the initial and final time periods, except for the 15% Lase Peroxide Lite Group activated with hybrid light [diode laser/blue LED (HL) (LPL15HL)], which showed no significant differences (Table 1). Another important fact that has already been described in the literature^23^ ^,^ ^27^ and corroborates with this study, is that the higher the concentration of hydrogen peroxide, the more acidic is the pH in the bleaching gels.

In this study, the EXP10VHL experimental group (gel with nitrogen-doped titanium oxide nanoparticles) activated with hybrid light [diode laser/violet LED (VHL) (experimental)], presented the highest pH values in comparison to the other groups evaluated. The HL and VHL hybrid light sources employed to activate the bleaching gels presented a minimum increase (less than 2°C) in pulp chamber temperature^1^, assuring effective dental bleaching with low pulp risk^6^. The values obtained for the pH of hydrogen peroxide, regardless of the group studied, remained above the level considered critical for enamel dissolution (pH 5.5 to 6.5)^21^. However, it was noted that as the concentration of the gel diminished, its pH increased (Table 1). Therefore, the pH of these bleaching gels is unlikely to cause severe morphological changes on the surface of the enamel.

Acid conditioning prior to bleaching is a procedure still used in dental practice, however, both its efficiency and damage to the tooth structure are still not clarified. A recent study^6^ showed that there is no difference in the color analysis when acid conditioning is previously done, added to this, this study showed an increased wear due to the acid conditioning when the same bleaching gel concentration was analyzed. Only the Group TBO35HL showed no increased wear in the area with acid etching, in comparison with the area that received only the bleaching gel (Table 2). In the comparison between groups, Group LPS25HL showed the highest wear for the area with acid etching (Table 2).

Comparing the control group, which underwent brushing cycles only, with the other groups, it was observed that the control group showed significantly less wear (Table 2) than the experimental groups in the area treated with both the acid etching and bleaching gel and the area treated only with bleaching gel. Certainly, these differences in wear are due to the changes caused by the bleaching agents on the dental enamel, results already found in the literature, in which tooth-bleaching techniques led to increased surface roughness and wear if subjected to simulated tooth brushing, when compared with the control group that was only brushed^13^ ^,^ ^16^ ^,^ ^23^.

Note that in this study the dental bleaching may have left the surface of the bovine enamel more susceptible to wear caused by the 100,000 simulated brushing cycles, which is in agreement with the findings in the literature, which claims that whitening makes the enamel surface more susceptible to abrasion and morphological changes^1^ ^,^ ^9^ ^,^ ^13^ ^,^ ^23^ ^,^ ^28^. The adopted simulated tooth brushing (100,000 simulated brushing cycles) caused wear between 12 μm and 13 μm that is not clinically significant because other factors contribute to the loss of this structure. These cycles are equivalent, on average, to two years of brushing *in vivo* ^4^ ^,^ ^15^, a period that was adopted for being considered as a safe interval between bleaching sessions.

The analysis of this research’s results, associated with the findings of the literature, allowed the evaluation that, in general, the 25% Lase Peroxide Sensy II Group activated with hybrid light (HL) (diode laser/blue LED) presented the highest wear values, causing greater aggression to the tooth structure. The acid etching was more severe in all groups, except for the 35% Total Blanc Office (HL) Group. Although a direct correlation was not found with the results of wear, it was observed that the lower the bleaching gel concentration, the more alkaline it tended to be.

Acid etching prior to bleaching can be indicated in clinical situations where there is difficulty in obtaining satisfactory results after attempts by conventional whitening techniques, especially in patients with a high degree of pigmentation, seeking a greater penetration of the gel to the enamel. However, few studies evaluate this procedure and its consequences, thus justifying the objective of this study, which, despite being an *in vitro* study, seeks to answer questions that may assist in the clinical practice of the procedure. The results of this study show that etching with 37% phosphoric acid can lead to increased enamel wear, and should not be used as a routine procedure, only when necessary in an attempt to avoid more invasive procedures such as cavity preparation and production of indirect restorations.

However, further studies related to the effects of acid etching, pH, and different concentrations of bleaching gels used in office are required to extrapolate the results to the clinical context.

## CONCLUSIONS

Within the limitations of this *in vitro* study, the following conclusions can be drawn:

There was a trend towards a decrease in the initial pH at the end, except in the Lase Peroxide Lite 15% Group. There was no direct correlation between the values of pH and surface wear.

Different bleaching gels are able to increase the surface wear values after simulated brushing, mainly in the Lase Peroxide Sensy 25% Group.

Acid etching before bleaching increased the surface wear values in all groups.
